# Clinical significance of germline breast cancer susceptibility gene (g*BRCA*) testing and olaparib as maintenance therapy for patients with pancreatic cancer

**DOI:** 10.1186/s12885-024-12722-8

**Published:** 2024-08-12

**Authors:** Yasuharu Kawamoto, Takuo Yamai, Kenji Ikezawa, Yusuke Seiki, Ko Watsuji, Takeru Hirao, Makiko Urabe, Yugo Kai, Ryoji Takada, Kaori Mukai, Tasuku Nakabori, Hiroyuki Uehara, Tazuko Inoue, Fumie Fujisawa, Kazuyoshi Ohkawa

**Affiliations:** 1 Department of Hepatobiliary and Pancreatic Oncology, Osaka International Cancer Institute, 3-1-69 Otemae, Chou-ku, Osaka, 541-8567 Japan; 2 Department of Gastroenterology, Osaka General Medical Center, Osaka, Japan; 3 Department of Genetic Oncology, Osaka International Cancer Institute, Osaka, Japan; 4 Department of Medical Oncology, Shiga General Hospital, Shiga, Japan

**Keywords:** BRACAnalysis, Family history, FOLFIRINOX, Hereditary Breast and Ovarian Cancer syndrome

## Abstract

**Background:**

Germline breast cancer susceptibility gene (g*BRCA*) mutation in patients with pancreatic cancer (PC) is not common in clinical practice. Therefore, factors that efficiently show g*BRCA* mutations and the real-world outcomes of olaparib maintenance therapy have not been fully established. In the present study, we clarified the indicators for the effective detection of g*BRCA* mutation and the efficacy and safety of olaparib as maintenance therapy.

**Methods:**

We retrospectively analyzed 84 patients with PC who underwent g*BRCA* testing (BRACAnalysis, Myriad Genetics, Salt Lake City, UT, USA) at our institute between January 2021 and March 2022. For each patient, clinical data were extracted from medical records.

**Results:**

The median patient age was 64 y (29–85 y), and 41 patients (48.8%) were male. The g*BRCA* mutations were identified in 10 (11.9%) patients; two patients had *BRCA1* mutation and eight had *BRCA2* mutation. All patients with g*BRCA* mutation had a family history of any cancer, and eight of them had a family history of Hereditary Breast and Ovarian Cancer syndrome (HBOC)-related cancer. The g*BRCA* mutation rate was higher for patients with PC with a family history of HBOC-related cancer compared to that in patients with PC having a family history of other cancers and no family history of cancer (22.9% vs. 4.1%; *P* = 0.014). In our study, eight out of 10 patients with g*BRCA*-positive PC received olaparib after platinum-based chemotherapy. The best responses to platinum-based chemotherapy included a complete response in one patient (12.5%) and a partial response in seven patients (87.5%). The median duration of treatment with platinum-based chemotherapy plus olaparib was 17.5 months (8–87 months), and the duration of treatment with olaparib maintenance therapy was 11 months (1–30 months). During olaparib maintenance therapy, three patients showed no disease progression. One of these three patients underwent conversion surgery after receiving olaparib for 12 months.

**Conclusions:**

The g*BRCA* testing should be considered proactively, especially in patients with PC with a family history of HBOC-related cancer.

## Background

Pancreatic cancer (PC) has the worst prognosis of all solid cancers [1]. Both the incidence and mortality of PC are increasing worldwide, including in the United States, Europe, and Japan, making it the third-most prevalent cause of cancer-related death in the United States [2]. Development of further treatment modalities is needed to improve prognosis [3–5]. PC has familial accumulation, and familial PC, defined as having two or more first-degree relatives (parents, siblings, or children) with PC, has been proposed [6]. There is increasing evidence for the genomic analysis of PC [7–10]. Germline pathological variants, such as *ATM*,* BRCA1*,* BRCA2*,* CHEK2*, and *PALB2*, which are known to cause homologous recombination deficiency (HRD), have been reported to be present in 10–20% of patients with PC [11–13]. Germline *BRCA1/2* (g*BRCA*) mutation is found in 4–7% of PC patients, occurring more frequently than other genes associated with HRD, such as *ATM*, *CHEK2*, and *PALB2* [14–19]. *BRCA1/2* encodes a tumor suppressor protein involved in the repair of double-stranded DNA breaks caused by homologous recombination defects [20, 21]. Mutations in g*BRCA* are associated with the risk of developing breast, ovarian, prostate, and pancreatic cancers [17, 22, 23]. Germline *BRCA* mutation, currently examined using BRACAnalysis (Myriad Genetics, Salt Lake City, UT, USA) in Japan, is principally implicated in Hereditary Breast and Ovarian Cancer syndrome (HBOC) [24, 25]. Once diagnosed with HBOC, regular screening for HBOC-related cancers can lead to early detection and treatment; risk-reducing surgeries are considered to be important treatment options [26–28].

The search for g*BRCA* as a biomarker for PC therapy is important in clinical practice. The prognosis of patients with g*BRCA*-positive PC is favorable, with 67.4–76.3% of these patients responding well to platinum-based chemotherapy [29, 30]. This is because g*BRCA*-mutant tumor cells assume that double-stranded DNA damage caused by platinum-based regimens could not be repaired due to HRD [31, 32]. The POLO study in 2019 investigated the efficacy of olaparib, a poly-ADP ribose polymerase (PARP) inhibitor, as maintenance therapy and indicated an improvement in median progression-free survival with olaparib maintenance therapy after platinum-based chemotherapy (7.4 months in the olaparib group vs. 3.8 months in the placebo group; hazard ratio for disease progression or death, 0.53; 95% confidence interval, 0.35–0.82; *P* = 0.004) [33]. Based on these results, olaparib as maintenance chemotherapy after platinum-based chemotherapy for patients with g*BRCA*-positive unresectable PC was covered by insurance in Japan in December 2020. BRACAnalysis was also approved by national insurance in Japan as a companion diagnostic test for PARP inhibitors in patients with PC [24]. The frequency of g*BRCA* mutation in patients with PC is uncommon in clinical practice. Hence, the factors that were associated with g*BRCA* mutation and the real-world outcomes of olaparib maintenance therapy have not been fully elucidated. The current study examined the indicators for the detection of g*BRCA* mutation and the efficacy and safety of olaparib maintenance therapy.

## Methods

### Patient identification and data collection

We conducted a retrospective, observational study of 84 patients with PC who underwent germline *BRCA1/2* genetic testing (BRACAnalysis) in a Japanese cancer referral center (Osaka International Cancer Institute) between January 2021 and March 2022. For each patient, clinical data were extracted from medical records. Follow-up data from the patients were censored on August 31, 2023. The following clinical parameters were obtained: age, sex, histological diagnosis, resectability (disease status) at the initiation of treatment for PC, and family history of any cancer. Resectability was discussed by the Cancer Board of our facility before initiating treatment and was classified as resectable (R), borderline-resectable (BR), locally-advanced unresectable (UR-LA), metastatic unresectable (UR-M), and postoperative recurrence according to the National Comprehensive Cancer Network guidelines [34]. The submission for BRACAnalysis was at the discretion of the physicians and the results were shown as either *BRCA1* or *BRCA2* mutations, variants of unknown significance, or no mutation.

Olaparib was used as maintenance therapy for patients without disease progression after platinum-based chemotherapy (≥ 16 weeks). Tumor responses were graded according to the Response Evaluation Criteria in Solid Tumor (RECIST) ver. 1.1 [35]. Treatment outcomes with olaparib as maintenance therapy were evaluated for the duration of olaparib treatment and the occurrence of adverse events (AEs). AEs were graded according to the National Cancer Institute Common Terminology Criteria for Adverse Events, version 5.0.

This study was conducted in accordance with the Declaration of Helsinki. Ethical approval was obtained from the Ethical Review Committee of the Osaka International Cancer Institute (20148-3). The requirement for informed consent was waived by the opt-out method of our hospital’s website.

### Statistical analysis

The relation between g*BRCA* mutation and family history was assessed using Fisher’s exact test. The duration of platinum-based chemotherapy plus olaparib and the duration of olaparib maintenance therapy were compared using Student’s *t*-test. Differences were considered statistically significant if *P* values were < 0.05. Statistical analysis was performed using EZR (Saitama Medical Center, Jichi Medical University, Saitama, Japan), a graphical interface for R, and the R Commander software package for Mac (version 1.61; R Foundation for Statistical Computing, Vienna, Austria) [36].

## Results

### Patient characteristics

The patient characteristics are summarized in Table 1. The median age was 64 y (29–85 y) and 41 patients (48.8%) were male. Seventy-nine patients (94.0%) were histologically diagnosed with adenocarcinoma, one patient (1.2%) with adenosquamous carcinoma, two patients (2.4%) with acinar cell carcinoma, and two patients (2.4%) with undifferentiated carcinoma. At treatment initiation for PC, two patients (2.4%) had BR, 22 patients (26.2%) had UR-LA, 45 patients (53.6%) had UR-M, and 15 patients (17.8%) had postoperative recurrence. Sixty-five patients (77.3%) had a family history of any cancer; of these, 35 patients (41.7%) had a family history of HBOC-related cancer, including 20 patients (23.8%) with breast cancer, 16 patients (19.0%) with PC, and 7 patients (8.3%) with prostate cancer. Thirty patients (35.7%) had a family history of cancer unrelated to HBOC, including gastric cancer and malignant lymphoma. For the first line chemotherapy, gemcitabine and nab-paclitaxel combination therapy was used in 46 patients (54.8%), FOLFIRINOX was used in 30 patients (35.7%), and other chemotherapeutic regimens were used in eight patients (9.5%).

**Table 1:** Characteristics of 84 patients with pancreatic cancer (PC) who underwent g*BRCA* testing (BRACAnalysis) at the Osaka International Cancer Institute between January 2021 and March 2022

Number of patients, *n*	84
Age, median (range), year	64 (29−85)
Male sex, n (%)	41 (48.8)
Histological diagnosis, n (%)	
adenocarcinoma	79 (94.0)
adenosquamous carcinoma	1 (1.2)
acinar cell carcinoma	2 (2.4)
undifferentiated carcinoma	2 (2.4)
Disease status at diagnosis, n (%)	
BR	2 (2.4)
UR-LA	22 (26.2)
UR-M	45 (53.6)
Postoperative recurrence	15 (17.8)
Family history of any cancer, n (%)	65 (77.3)
HBOC-related cancer	35 (41.7)
Breast cancer	20 (23.8)
Ovarian cancer	0 (0.0)
Pancreatic cancer	16 (19.0)
Prostate cancer	7 (8.3)
HBOC-unrelated cancer	30 (35.7)

BR, borderline-resectable; UR-LA, locally-advanced unresectable; UR-M, metastatic unresectable; HBOC, hereditary breast and ovarian cancer syndrome

### Results of g*BRCA* testing

The results of g*BRCA* testing (BRACAnalysis) are summarized in Table 2. The timing of undergoing g*BRCA* testing was: 12 patients (14.3%) underwent g*BRCA* testing before the initiation of chemotherapy, 50 patients (59.5%) during first line treatment, 18 patients (21.4%) during second line treatment, and four patients (4.8%) after third line treatment. Germline *BRCA* mutation was identified in 10 patients (11.9%). Two of them had germline *BRCA1* mutation and eight had germline *BRCA2* mutation. All patients with g*BRCA*-positive PC had a family history of any cancer, and eight of them had a family history of HBOC-related cancer. The g*BRCA* mutation rate was higher for patients with PC with a family history of HBOC-related cancer than in patients with PC with a family history of other cancer or no family history of cancer (22.9% vs. 4.1%; *P* = 0.014). Genetic counseling was recommended to all patients with g*BRCA*-positive PC and eight received counseling.

**Table 2:** Results of g*BRCA* testing

The timing of undergoing g*BRCA* testing, *n* (%)			
before the initiation of chemotherapy	12 (14.3)		
during first line chemotherapy	50 (59.5)		
during second line chemotherapy	18 (21.4)		
after third line chemotherapy	4 (4.8)		
The number of g*BRCA* mutation patients, n	10		
*BRCA1* mutation	2		
*BRCA2* mutation	8		
The relation between g*BRCA* mutation and family history, n (%)			
	g*BRCA* mutation		*P* value*
	positive	negative	
Family history			0.014
HBOC-related cancer (*n* = 35)	8 (22.9)	27 (77.1)	
Other cancer or no family history of cancer (*n* = 49)	2 (4.1)	47 (95.9)	

* Fisher’s exact test

BRCA, breast cancer susceptibility gene; HBOC, Hereditary Breast and Ovarian Cancer

### Treatment outcomes in patients with g*BRCA*-positive PC

In this study, all ten patients with g*BRCA*-positive PC received platinum-based chemotherapy. Among them, eight patients switched to olaparib maintenance therapy, one patient experienced tumor progression before switching to olaparib, and one underwent conversion surgery due to excellent response to FOLFIRINOX.

The treatment outcomes of patients who underwent platinum-based chemotherapy and olaparib maintenance therapy are shown in Table 3. The number of chemotherapeutic regimens before initiating platinum-based chemotherapy was zero in five patients (62.5%), one in two patients (25.0%), and two in one patient (12.5%). The median duration of platinum-based chemotherapy was 9 months (4–57 months). Of the eight patients who underwent olaparib maintenance therapy, the best responses to platinum-based chemotherapy included a complete response in one patient (12.5%) and a partial response in seven patients (87.5%). The median duration of treatment with platinum-based chemotherapy plus olaparib was 17.5 months (8–87 months), and the duration of treatment with olaparib maintenance therapy was 11 months (1–30 months). Between five patients who underwent BRACAnalysis at an earlier timing (before the initiation of chemotherapy and during first line) and three patients at a later timing (during second line or later), there was no significant difference between the duration of platinum-based chemotherapy plus olaparib (median (range); 14 months (8–37 months) vs. 28 months (15–87 months); *P* = 0.49), and the duration of olaparib maintenance therapy (median (range); 9 months (1–30 months) vs. 12 months (10–12 months); *P* = 0.46).

During olaparib maintenance therapy, five out of eight patients were judged to have progressive disease, leading to the discontinuation of olaparib. Four of these five patients subsequently underwent different chemotherapeutic regimens, while the other patient chose not to continue further chemotherapy due to advanced age. The remaining three of the eight patients were free from disease progression. One of these three patients underwent conversion surgery after receiving olaparib for 12 months and the other two patients continued to receive olaparib at the end of follow-up.

**Table 3:** Treatment outcomes of platinum-based chemotherapy and olaparib maintenance therapy in patients with g*BRCA*-positive pancreatic cancer (PC)

Patients with g*BRCA*-positive PC, *n*	10
Patients who underwent olaparib maintenance therapy, n (%)	8 (80.0)
Chemotherapeutic regimens before initiating platinum-based chemotherapy, n (%)	
0	5 (62.5)
1	2 (25.0)
2	1 (12.5)
Duration of platinum-based chemotherapy, median (range), month	9 (4−57)
Best response of platinum-based chemotherapy before olaparib maintenance therapy, n (%)	
Complete response	1 (12.5)
Partial response	7 (87.5)
Stable disease	0 (0)
Progression of disease	0 (0)
Duration of platinum-based chemotherapy and olaparib maintenance therapy, median (range), month	17.5 (8−87)
Duration of olaparib maintenance therapy, median (range), month	11.0 (1−30)

BRCA, breast cancer susceptibility gene

The AEs during the entire period of olaparib maintenance therapy are shown in Table 4. The most common AEs (all grades) were fatigue, nausea, and anemia. Grade 3 or higher AEs were anemia and thrombocytopenia in one patient, and AEs were managed by dose modification.

**Table 4:** Summary of adverse events during the entire period of olaparib maintenance therapy

Adverse events (AEs), *n* (%)	Any Grade	Grade ≥ 3
Any	7 (87.5)	1 (12.5)
Fatigue	3 (37.5)	0 (0)
Nausea	2 (25.0)	0 (0)
Anemia	2 (25.0)	1 (12.5)
Thrombocytopenia	1 (12.5)	1 (12.5)
Abdominal pain	0 (0)	0 (0)
Diarrhea	1 (12.5)	0 (0)
Decreased appetite	0 (0)	0 (0)
Constipation	0 (0)	0 (0)
Vomiting	0 (0)	0 (0)
Oral mucositis	1 (12.5)	0 (0)

## Discussion

In the current study, 11.9% of patients with PC were g*BRCA* positive. The frequency of g*BRCA* mutation has been reported to be 4–7% in patients with PC [14–19]. The frequency of the g*BRCA* mutation and g*BRCA*-positive rates in the current study were higher than that in previous reports. The selection of patients for g*BRCA* testing was done at the discretion of each physician, which may have strongly influenced the high positive rate. Furthermore, g*BRCA* mutation was detected in 22.9% of patients with PC with a family history of HBOC-related cancer, compared to only 4.1% of patients with PC with no family history of HBOC-related cancer. A family history of any cancer has been reported in 94.4% of patients with g*BRCA*-positive PC, of which 79.6% had a family history of HBOC-related cancer [37]. A substantial difference in the frequency of a family history of HBOC-related cancer has been clarified between patient groups with g*BRCA* mutation-positive and -negative PC [19]. Taken together with these previous results, a family history of HBOC-related cancer could be an indicator for the detection of g*BRCA* mutation. Since approximately 4% of patients without a family history of HBOC-related cancer were g*BRCA*-positive, g*BRCA* testing should also be considered for these patients as well.

The timing for undergoing g*BRCA* testing in this study (before the initiation of chemotherapy, 14.3%; during first line chemotherapy, 59.5%) was earlier than that in a previous report (before the initiation of chemotherapy, 17.6%; during first line chemotherapy, 36.8%) [19]. The results of g*BRCA* testing could play an important role in the selection of a second-line chemotherapeutic regimen because platinum-based chemotherapy, such as FOLFIRINOX, is preferred because it shows favorable treatment outcomes in patients with g*BRCA*-positive PC [38–41]. This preference may influence physicians’ decisions regarding the timing of g*BRCA* testing. Early submission of g*BRCA* testing before the end of first-line chemotherapy is considered important, especially when gemcitabine and nab-paclitaxel combination therapy is chosen as the first-line chemotherapy.

In the current study, the median duration of olaparib maintenance therapy was 11 months, including the case in which conversion surgery was performed. Long-term combination chemotherapy for patients with unresectable PC has been reported to endorse eligibility for conversion surgery [42–44]. In a study of patients with unresectable PC treated with combination chemotherapy, chemotherapy for > 8 months was reported to be an independent prognostic factor [45]. Therefore, long-term continuation of chemotherapy with tolerable toxicity is important for patients with unresectable PC. However, long-term administration of FOLFIRINOX can cause various AEs, including chemotherapy-induced peripheral neuropathy (CIPN), which is primarily caused by oxaliplatin [46–48]. Severe CIPN can affect the continuation of chemotherapy and patient prognosis [49]. Olaparib maintenance therapy can be a useful treatment option with manageable toxicity, especially in patients with severe CIPN [50]. Recently, Stossel et al. reported that patients with g*BRCA*-positive PC had a favorable response to platinum-based chemotherapy and PARP inhibitors, but most patients experienced resistance [51]. Homologous recombination proficiency and secondary mutations restoring partial functionality have been identified as the dominant mechanisms of resistance. Further research is required to address the treatment strategies for patients who develop resistance.

This study had some limitations. First, this was a single-center, retrospective, observational study. Second, the selection of patients for whom g*BRCA* testing was performed, the timing of g*BRCA* testing, and the timing of transition to olaparib maintenance therapy in patients with g*BRCA*-positive PC were at the discretion of each physician. The limited number of patients may have introduced selection bias.

## Conclusion

In our single-center, retrospective study, g*BRCA* mutation was particularly high in patients with PC with a family history of HBOC-related cancer. Platinum-based chemotherapy and olaparib maintenance therapy for patients with g*BRCA*-positive PC can be expected to prolong the prognosis; therefore, g*BRCA* testing should be considered proactively, especially for patients with PC with a family history of HBOC-related cancer.

## Data Availability

The datasets used and/or analyzed during the current study are available from the corresponding author on reasonable request. The data that support the findings of this study are available on reasonable request from the corresponding author.

## Abbreviations

AEs: Adverse events
BR: Borderline-resectable
CIPN: Chemotherapy-induced peripheral neuropathy
g*BRCA*: Germline breast cancer susceptibility gene
HBOC: Hereditary Breast and Ovarian Cancer syndrome
HRD: Homologous recombination deficiency
PARP: Poly-ADP ribose polymerase
PC: Pancreatic cancer
R: Resectable
RECIST: Response Evaluation Criteria in Solid Tumor
UR-LA: Locally-advanced unresectable
UR-M: Metastatic unresectable
